# Identification of core genes in the progression of endometrial cancer and cancer cell-derived exosomes by an integrative analysis

**DOI:** 10.1038/s41598-020-66872-3

**Published:** 2020-06-17

**Authors:** Shuang Shi, Qiang Tan, Fuqiang Feng, Heping Huang, Jingjie Liang, Dingren Cao, Zhengguang Wang

**Affiliations:** 1 College of Animal Sciences, Zhejiang University, Hangzhou, Zhejiang P. R. China; 2 Agricultural Economic Service Center of Wuzhen Town, Tongxiang, Zhejiang P. R. China

**Keywords:** Cancer, Cell biology, Biomarkers

## Abstract

Endometrial cancer is one of the most prevalent tumors of the female reproductive system causing serious health effects to women worldwide. Although numerous studies, including analysis of gene expression profile and cellular microenvironment have been reported in this field, pathogenesis of this disease remains unclear. In this study, we performed a system bioinformatics analysis of endometrial cancer using the Gene Expression Omnibus (GEO) datasets (GSE17025, GSE63678, and GSE115810) to identify the core genes. In addition, exosomes derived from endometrial cancer cells were also isolated and identified. First, we analyzed the differentially expressed genes (DEGs) between endometrial cancer tissues and normal tissues in clinic samples. We found that HAND2-AS1, PEG3, OGN, SFRP4, and OSR2 were co-expressed across all 3 datasets. Pathways analysis showed that several pathways associated with endometrial cancer, including “p53 signaling pathway”, “Glutathione metabolism”, “Cell cycle”, and etc. Next, we selected DEGs with highly significant fold change and co-expressed across the 3 datasets and validated them in the TCGA database using Gene Expression Profiling Interactive Analysis (GEPIA). Finally, we performed a survival analysis and identified four genes (TOP2A, ASPM, EFEMP1, and FOXL2) that play key roles in endometrial cancer. We found up-regulation of TOP2A and ASPM in endometrial cancer tissues or cells, while EFEMP1 and FOXL2 were down-regulated. Furthermore, we isolated exosomes from the culturing supernatants of endometrial cancer cells (Ishikawa and HEC-1-A) and found that miR-133a, which regulates expression of FOXL2, were present in exosomes and that they could be delivered to normal endometrial cells. The common DEGs, pathways, and exosomal miRNAs identified in this study might play an important role in progression as well as diagnosis of endometrial cancer. In conclusion, our results provide insights into the pathogenesis and risk assessment of endometrial cancer. Even so, further studies are required to elucidate on the precise mechanism of action of these genes in endometrial cancer.

## Introduction

Endometrial cancer, a gynecologic malignancy tumor ranked behind breast, lung, and colorectal cancers^1^, greatly affects women’s health around the world. In the recent past, incidence and the rate of this disease has increased due to lack of effective diagnosis before menopause^2^. Meanwhile, patient age at disease onset has also decreased due to changes in lifestyles^3^. In general, endometrial cancer is classified into two types based on pathological and histological characteristics^2^. Type I commonly known as endometrioid adenocarcinoma is the major type of endometrial cancer^4^. Type II is the second category of endometrial cancer, and have a higher prevalence and poor prognosis^5^. Early diagnosis is required for effective disease assessment and treatment, but numerous studies are now focusing on specific molecular alterations that aid in the development of endometrial cancers^6,7^.

Initiation and progression of tumors is associated with abnormal expression of genes and the cancer microenvironment^8^. Numerous processes and factors have been shown to medicate cell-cell and cell-microenvironment communication. Most importantly, tumor cells secrete molecules to the extracellular microenvironment, and these contribute to cancer progression. In addition, these molecules are regarded as biomarkers for diagnosis of diseases. Recent studies have demonstrated that exosomes, with the size range from 40 to 200 nm, which contain cargos including proteins, lipids, and nucleic acids^9^, transfer signaling molecules to cells via the extracellular environment. This is a novel mode of cellular communication that could influence the physiological conditions and behaviors of the recipient cells, and also connect with a variety of diseases process^10–12^. miRNAs, a group of small non-coding RNAs that regulate expression of endogenous genes by targeting to 3’ untranslated region (3' UTR)^13^, are mainly present in exosomes and play essential roles in occurrence of multiple tumor types^14,15^. Exosomal miRNAs mediate regulation of cancer milieu, containing cancer metastasis and progression^16^. In addition, exosomes also serve as a source of diagnostic and prognostic biomarkers for endometrial cancer^12,17^. Integrative research on endometrial cancer is still lacking with most studies only focusing on gene expression or exosomal cargos of the disease^18–20^.

Gene chips analysis is a useful tool for identifying the factors and genes involved in endometrial cancer^21^. Previous studies, particularly those focusing on screening differentially expressed genes, have reported identification of several targets and elucidated the roles played by specific biological process and pathways in endometrial cancer^22,23^. In this study, an integrative bioinformatics analysis was performed on endometrial cancer and normal endometrial tissues. Using the Gene Expression Omnibus (GEO) database, several key differentially expressed genes (DEGs) were identified, and their biological processes and pathways found to be closely associated with endometrial cancer. In addition, we found that endometrial cancer cells-derived exosomal miRNA targets the core DEG. These exosomes also could be delivered to normal cells.

## Results

### Identification of DEGs in endometrial cancer

**Figure 1:**
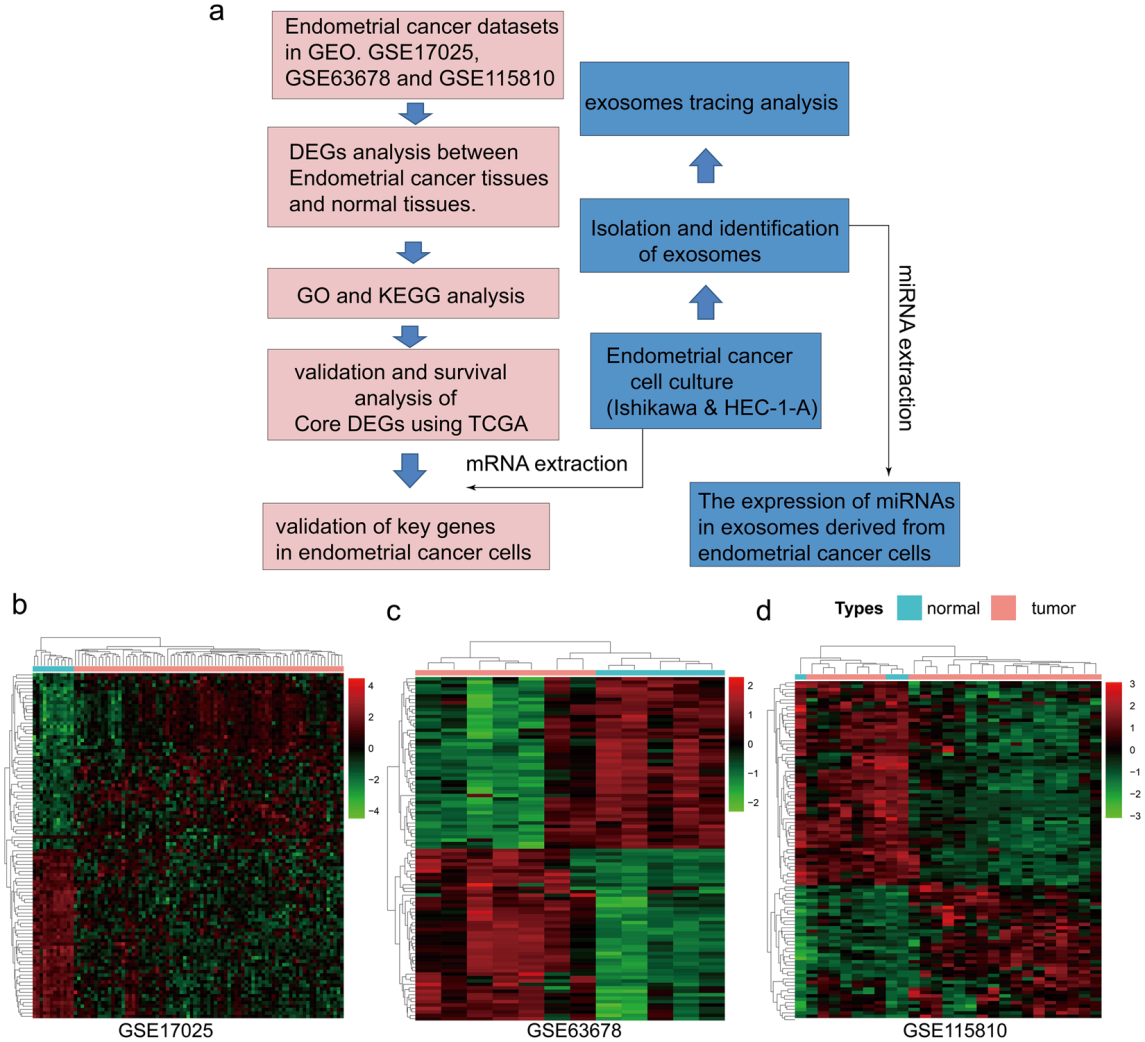
(**a**) Flow diagram of the study design. GO, gene ontology. KEGG, Kyoto Encyclopedia of Genes and Genomes. (**b–d**) Heat maps showed the DEGs between normal endometrial tissues and endometrial cancer tissue in three GEO datasets. Heat map for potential DEGs in GSE17025 (containing 12 normal endometrial tissues and 79 tumor tissues) (**b**) GSE63678 (containing 5 normal endometrial tissues and 7 tumor tissues) (**c**) GSE115810 (containing 3 normal endometrial tissues and 24 tumor tissues) (**d**).

**Figure 2:**
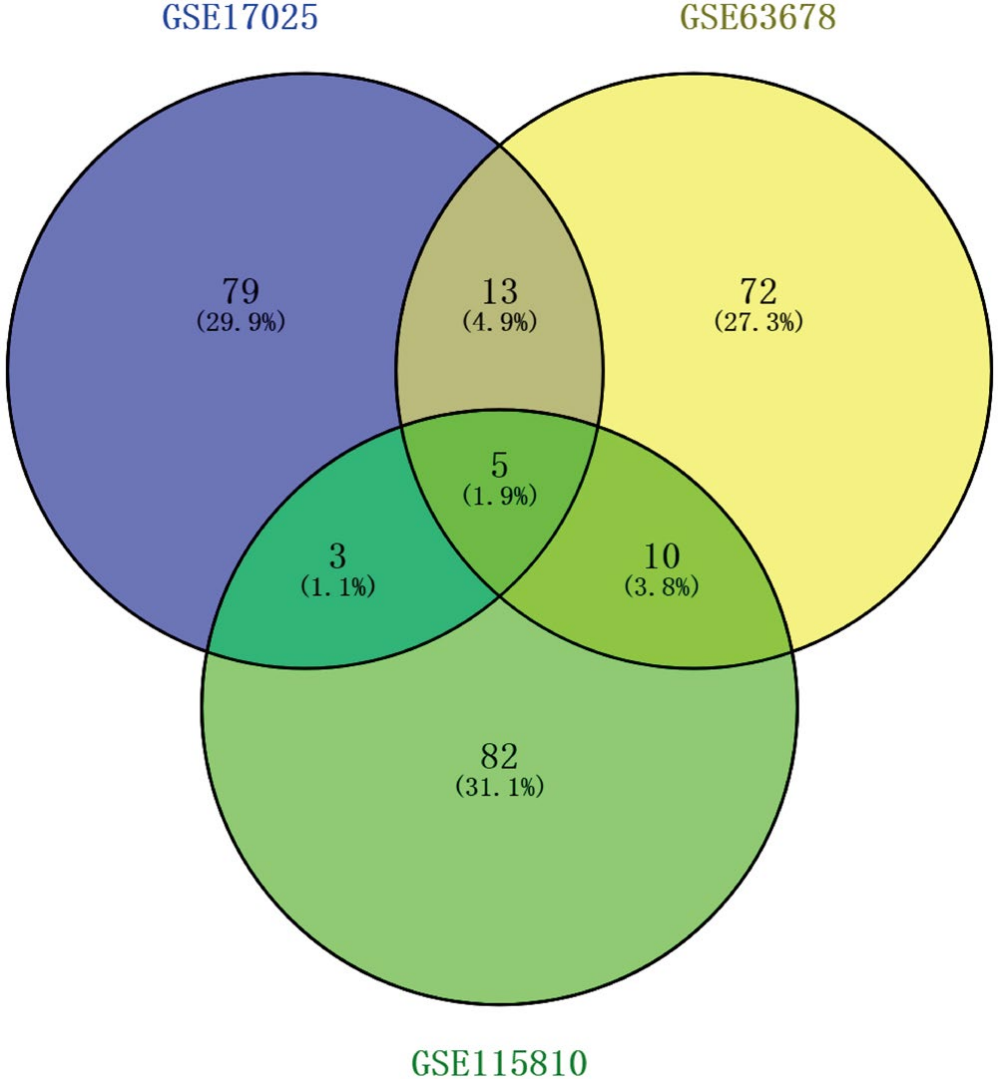
The Venn diagram shows the top 100 DEGs and co-expressed genes among GSE17025, GSE63678 and GSE115810.

We used basic packages implemented in R to identify potential DEGs across the three data sets. Based on a cut-off criteria of adjust P-value <0.05 and |logFC | >1.0, we identified a total of 2346, 340, and 233 genes in GSE17025, GSE63678 and GSE115810, respectively. The top 10 DEGs with the largest fold changes in the three GEO data sets are shown in Tables 1–3, while the top 100 DEGs in each dataset, represented by heat maps are shown in Fig. 1b–d. After expurgating duplicate genes and expression values which lacked specific gene symbols, we combined the top 100 DEGs in GSE17025, GSE63678 and GSE115810 to create a Venn diagram. Five genes were found to be co-expressed across all three data sets, including HAND2-AS1, PEG3, OGN, SFRP4, and OSR2. All these genes were, however, down-regulated in endometrial cancer tissues (Fig. 2). Three genes, including FOSB, FOS and LTBP4, were co-expressed in GSE17025 and GSE115810. In addition, a total of 10 genes, including CFB, RBM47, WT1, ADAMTS5, TSPYL5, HTR2B, ANK2, FOXL2, CXCL12 and EFEMP1, were co-expressed in GSE63678 and GSE115810. On the other hand, 13 genes, including BCHE, LMOD1, TPX2, CXCL9, LIPG, TFAP2A, DLGAP5, MMP12, CEP55, RRM2, MELK, TOP2A and ASPM were co-expressed in GSE17025 and GSE63678,.

**Table 1:** Top 10 differentially expressed genes in GSE17025.

Rank	Probe set ID	Gene symbol	Description	Regulation
1	202768_at	FOSB	FBJ osteosarcoma oncogene B	Down-regulation
2	206622_at	TRH	thyrotropin releasing hormone	Down-regulation
3	219791_s_at	HAND2-AS1	HAND2 antisense RNA 1	Down-regulation
4	206228_at	PAX2	paired box 2	Down-regulation
5	209242_at	PEG3	paternally expressed 3	Down-regulation
6	201291_s_at	TOP2A	DNA topoisomerase II alpha	Up-regulation
7	1553655_at	CDC20B	cell division cycle 20B	Up-regulation
8	219918_s_at	ASPM	abnormal spindle microtubule assembly	Up-regulation
9	202018_s_at	LTF	lactotransferrin	Up-regulation
10	205476_at	CCL20	C-C motif chemokine ligand 20	Up-regulation

**Table 2:** Top 10 differentially expressed genes in GSE63678.

Rank	Probe set ID	Gene symbol	Description	Regulation
1	209242_at	PEG3	paternally expressed 3	Down-regulation
2	219791_s_at	HAND2-AS1	HAND2 antisense RNA 1	Down-regulation
3	206067_s_at	WT1	WT1 transcription factor	Down-regulation
4	205433_at	BCHE	butyrylcholinesterase	Down-regulation
5	218730_s_at	OGN	osteoglycin	Down-regulation
6	204653_at	TFAP2A	transcription factor AP-2 alpha	Up-regulation
7	201839_s_at	EPCAM	epithelial cell adhesion molecule	Up-regulation
8	201596_x_at	KRT18	keratin 18	Up-regulation
9	219148_at	PBK	PDZ binding kinase	Up-regulation
10	204580_at	MMP12	matrix metallopeptidase 12	Up-regulation

**Table 3:** Top 10 differentially expressed genes in GSE115810.

Rank	Probe set ID	Gene symbol	Description	Regulation
1	204051_s_at	SFRP4	secreted frizzled related protein 4	Down-regulation
2	219791_s_at	HAND2-AS1	HAND2 antisense RNA 1	Down-regulation
3	220102_at	FOXL2	forkhead box L2	Down-regulation
4	201842_s_at	EFEMP1	EGF containing fibulin extracellular matrix protein 1	Down-regulation
5	206637_at	P2RY14	purinergic receptor P2Y14	Down-regulation
6	201195_s_at	SLC7A5	solute carrier family 7 member 5	Up-regulation
7	202357_s_at	CFB	complement factor B	Up-regulation
8	209875_s_at	SPP1	secreted phosphoprotein 1	Up-regulation
9	204846_at	CP	ceruloplasmin	Up-regulation
10	204623_at	TFF3	trefoil factor 3	Up-regulation

### Biological process analysis of DEGs

**Figure 3:**
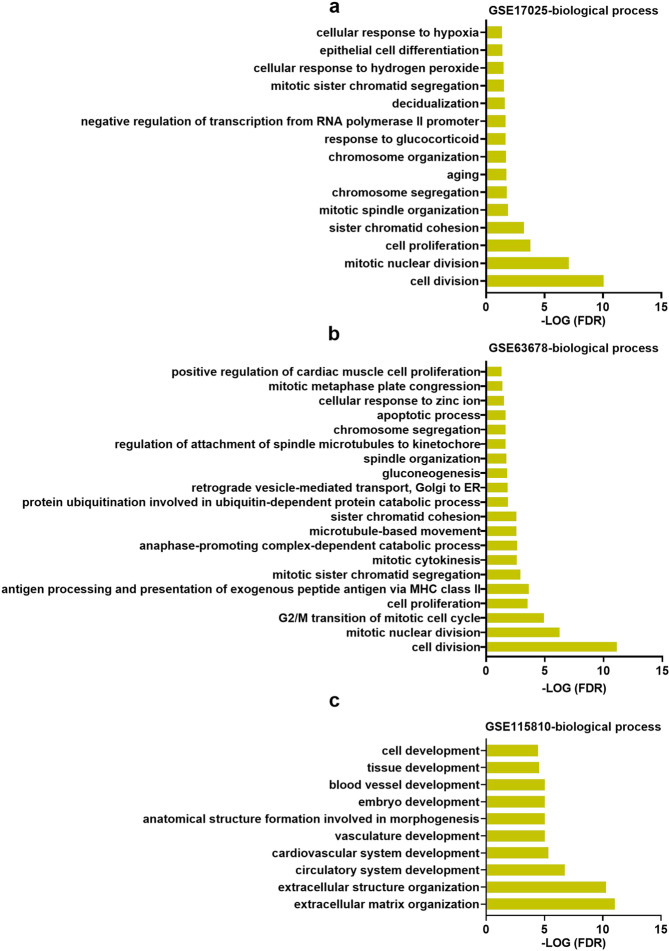
The significant biological functions in terms of GO analysis related to DEGs in GSE17025 (**a**), GSE63678 (**b**) and GSE115810 (**c**).

To gain more insights into the function of the DEGs, we performed a GO analysis. Sorting the transcripts by false discovery rate (FDR) revealed that there are 15, 20 and 10 GO terms of biological processes in GSE17025, GSE63678 and GSE115810, respectively (Fig. 3a–c). There was no common biological processes were found across the 3 datasets. Nevertheless, we found 6 common biological processes including “ cell division “, “mitotic nuclear division”, “ cell proliferation “, “ sister chromatid cohesion “, “ chromosome segregation “, and “mitotic sister chromatid segregation” in GSE63678 and GSE17025. Other biological processes, such as “epithelial cell differentiation”, “apoptotic process “, “extracellular matrix organization”, “ cell development “, “blood vessel development” and “ extracellular structure organization “, were also identified very vital in each dataset.

### Signaling pathway enrichment analysis

**Figure 4:**
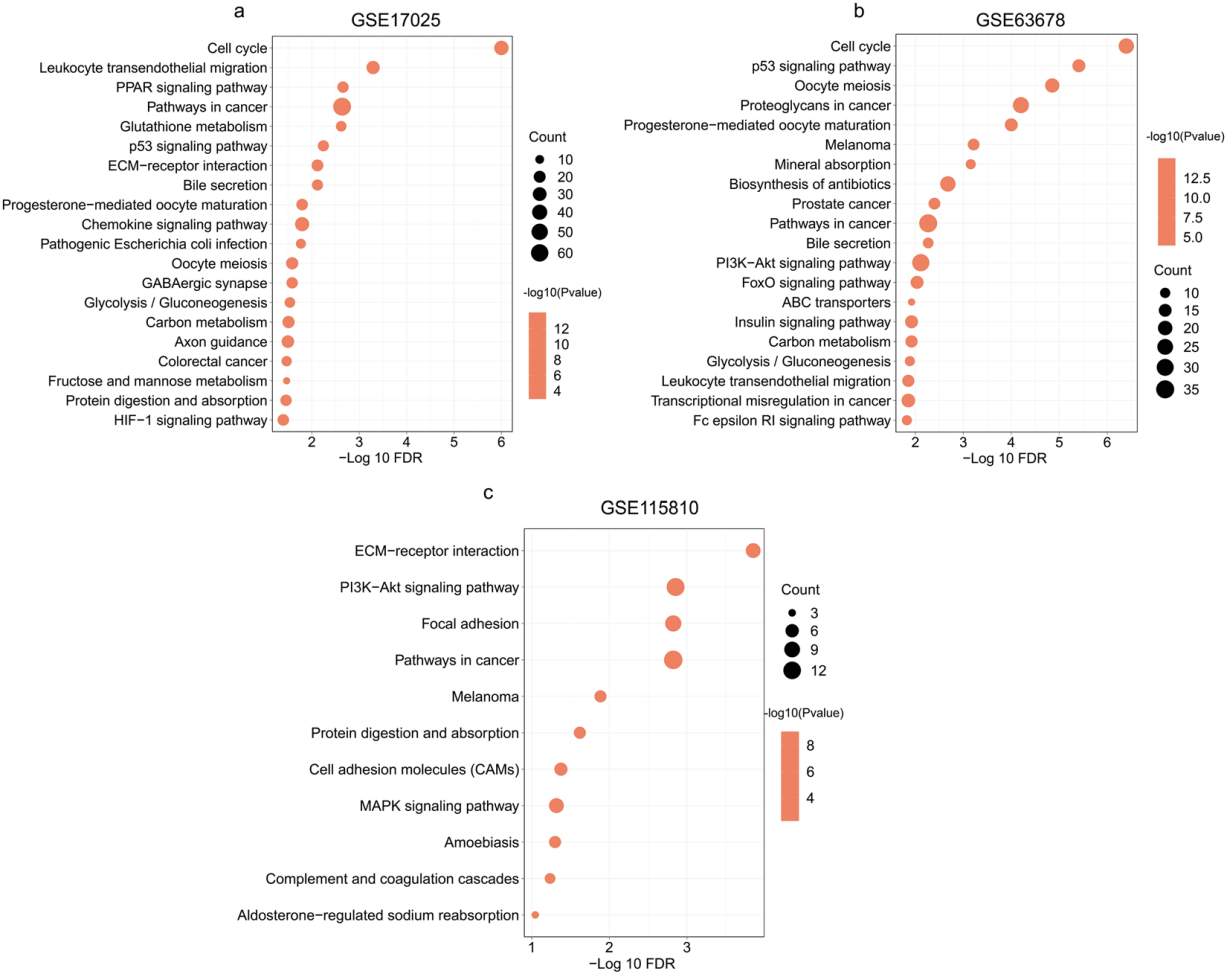
Pathways in terms of KEGG pathway analysis related to DEGs in GSE17025 (**a**), GSE63678 (**b**) and GSE115810 (**c**).

The potential pathways regulated by the DEGs in endometrial cancer were explored using KEGG analysis, We found that specific pathways were enriched in each dataset (Fig. 4a–c). Bubble plots from this analysis presented the most significantly enriched pathways (Fig. 4a–c). Among them, “Pathways in cancer” was the common pathway in the three datasets. The top 10 pathways are as outlined in Table 4. Of these, 7 pathways, including those involved in p53 signaling pathway, Glutathione metabolism, Cell cycle, ECM-receptor interaction, PI3K-Akt signaling pathway, Focal adhesion and MAPK signaling were notable because of their association with cancer progression. Particularly, these pathways were implicated in processes such as cell proliferation, cell migration and invasion. Pathways involved in HIF-1 signaling pathway, protein digestion and absorption as well as FoxO signaling pathway were also other interesting pathways following pairwise comparisons.

**Table 4:** Top 10 pathways in pathway relation network of the 3 datasets (GSE17025, GSE63678 and GSE115810).

Rank	GSE17025	GSE63678	GSE115810
1	Cell cycle	Cell cycle	ECM-receptor interaction
2	Leukocyte transendothelial migration	p53 signaling pathway	PI3K-Akt signaling pathway
3	PPAR signaling pathway	Oocyte meiosis	Focal adhesion
4	Pathways in cancer	Proteoglycans in cancer	Pathways in cancer
5	Glutathione metabolism	Progesterone-mediated oocyte maturation	Melanoma
6	p53 signaling pathway	Melanoma	Protein digestion and absorption
7	ECM-receptor interaction	Mineral absorption	Cell adhesion molecules (CAMs)
8	Bile secretion	Biosynthesis of antibiotics	MAPK signaling pathway
9	Progesterone-mediated oocyte maturation	Prostate cancer	Amoebiasis
10	Chemokine signaling pathway	Pathways in cancer	Complement and coagulation cascades

### Validation of DEGs in TCGA database

**Figure 5:**
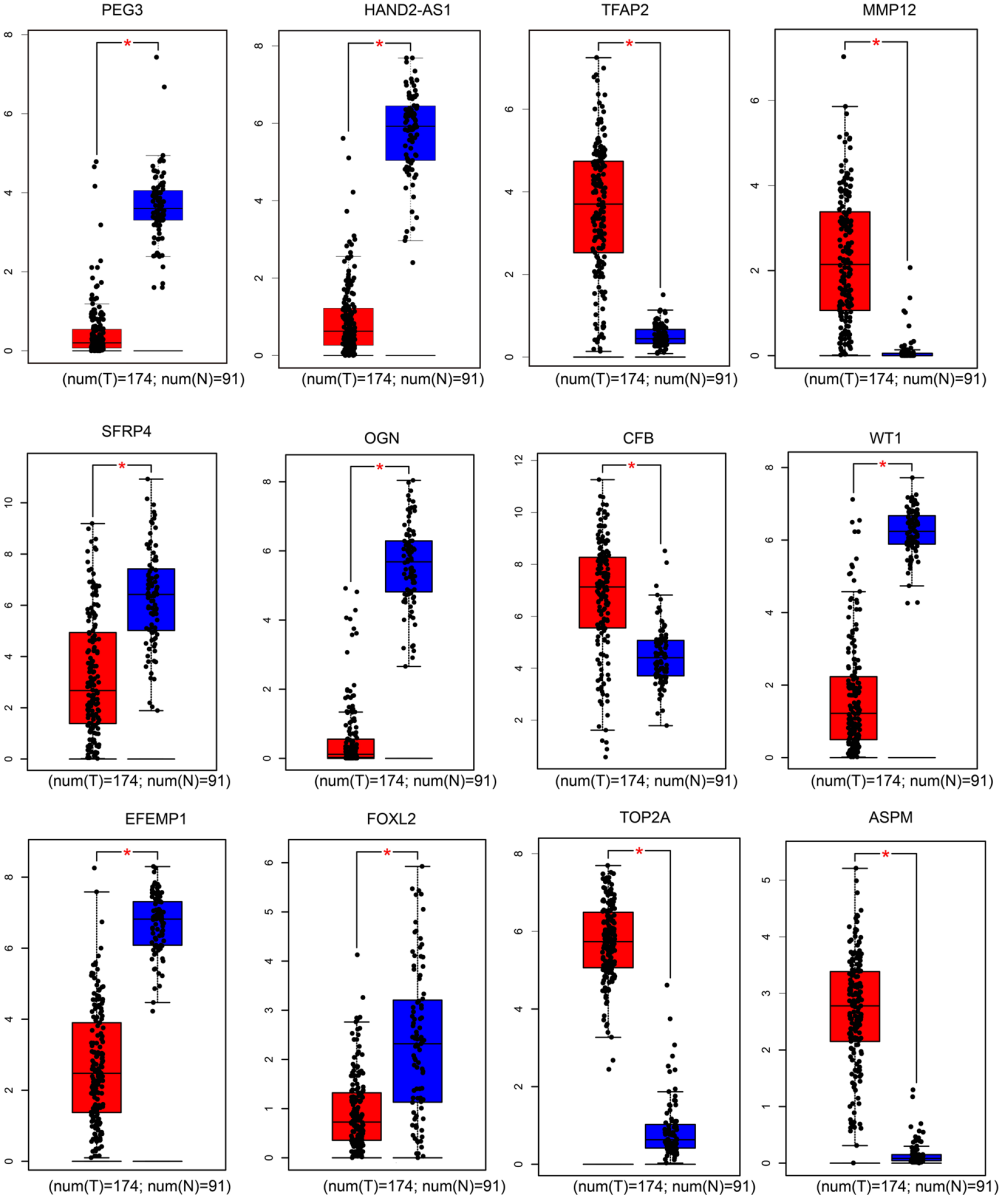
The GEPIA was used to verify the DEGs between normal endometrial tissues and endometrial cancer tissues in the TCGA database.

To further validate and determine the core genes highly associate with endometrial cancer from these DEGs, eight down-regulated and five up-regulated genes that showed the most significant fold changes (top10) between endometrial cancer and normal tissue in GSE17025, GSE63678 and GSE115810, were selected. Meanwhile, these 13 genes are derived from DEGs that overlap across the three data sets (Fig. 2). These included HAND2-AS1, PEG3, OGN, SFRP4, TFAP2A, MMP12, TOP2A, ASPM, FOSB, CFB, WT1, FOXL2 and EFEMP1. Hypothesis test analysis proves that these 13 genes have statistical significance (data not shown). Next, we explored the Gene Expression Profiling Interactive Analysis (GEPIA) database for identification and retrieval of the expression profiles of these DEGs in both endometrial cancer and normal tissues. We did not find any significant differences in FOSB expression in the GEPIA database between the treatments. However, expression profiles of the remaining twelve DEGs were consistent with our analysis results in GEO database. Particularly, expression of TFAP2A, MMP12, TOP2A, ASPM and CFB were higher in endometrial cancer relative to normal tissues. On the other hand, HAND2-AS1, PEG3, OGN, SFRP4, WT1, FOXL2 and EFEMP1 were significantly lower in endometrial cancer tissues than in normal tissues (Fig. 5).

### Survival analysis of core genes

**Figure 6:**
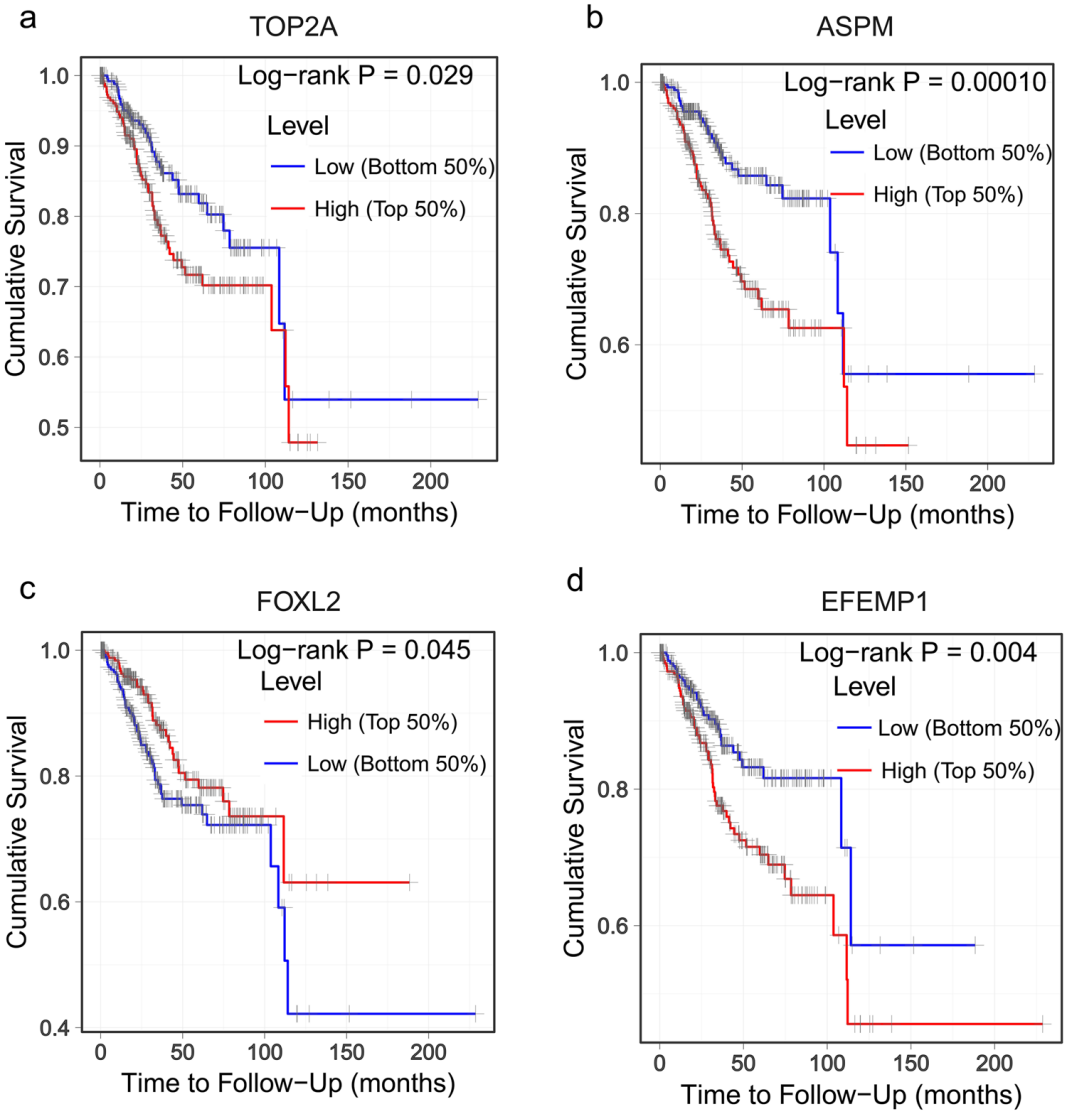
Survival analysis of the core genes (**a**) TOP2A, (**b**) ASPM, (**c**) FOXL2, (**d**) EFEMP1.

We then used prognostic information of the 12 core genes, available in the free online TIMER database (Tumor IMmune Estimation Resource) (https://cistrome.shinyapps.io/timer/), to perform a survival analysis. Aberrant expression levels of four genes out of 12 core DEGs showed a significant (P < 0.05)association with poor prognosis of endometrial cancer, and this included two up-regulated (TOP2A and ASPM) and two down-regulated (FOXL2 and EFEMP1) genes (Fig. 6a–d). This indicated that the four genes might play important roles in the progression of endometrial cancer.

### Validation of core genes expression in endometrial cancer cells

**Figure 7:**
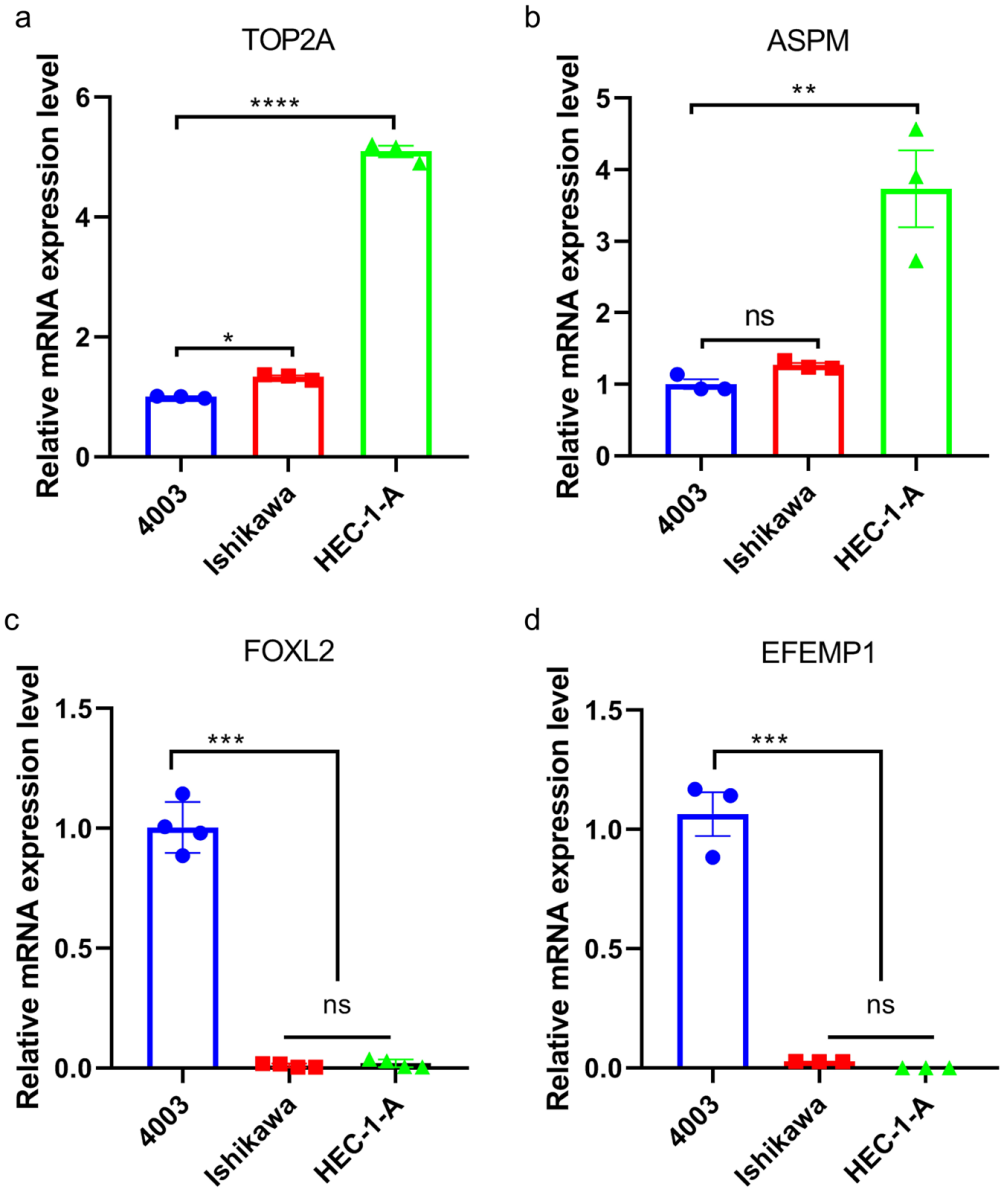
The validation of four core DEGs in endometrial cell lines. (**a**) TOP2A. (**b**) ASPM, (**c**) FOXL2, (**d**) EFEMP1.

Given the lack of clinical samples, we only used three cell lines in this study to validate the results of endometrial cancer from bioinformatics analyses. Ishikawa and HEC-1-A cell lines were derived from endometrioid adenocarcinoma, while 4003 was the normal endometrial cells and was used as a control. Real-time qPCR assay was employed to quantify expression levels of 4(TOP2A, ASPM, EFEMP1, and FOXL2) core DEGs across the three types of cells. Results showed a significant down-regulation of EFEMP1 and FOXL2 in endometrial cancer cells (Fig. 7c,d). A comparison with normal cells showed that TOP2A was highly expressed in endometrial cancer cells (Fig. 7a), which was consistent with the resultsfrom bioinformatics analysis above. However, we noted that ASPM was only significantly expressed in HEC-1-A cells (Fig. 7b).

### Exosomes secretion by endometrial cancer cells

**Figure 8:**
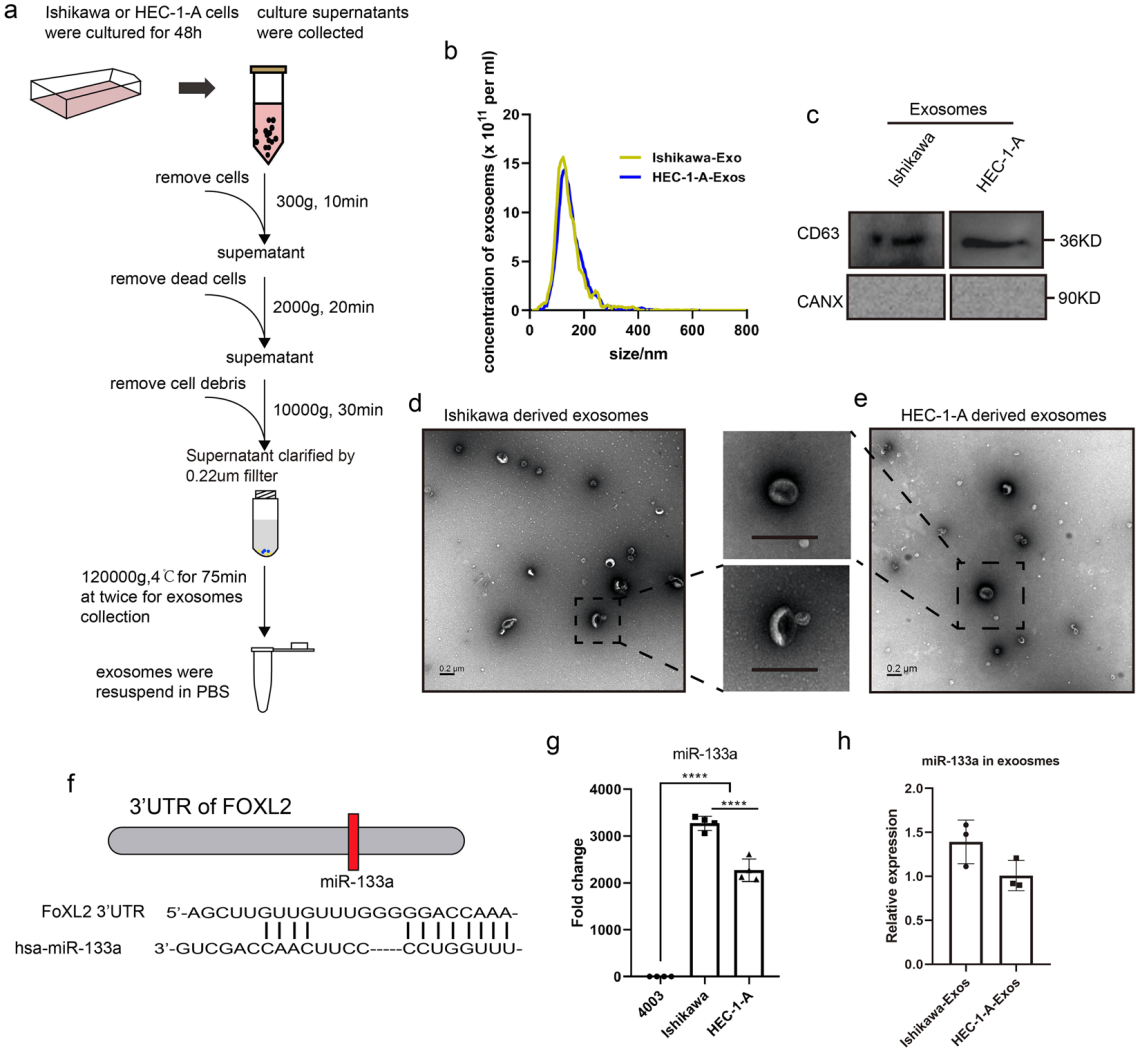
Endometrial cancer cells secret exosomal miRNAs. (**a**) Procedure for isolating exosomes from Ishikawa and HEC-1-A cell culture supernatant using ultracentrifugation. (**b**) NTA analysis of exosomes size distribution and concentration. (**c**) Western blot analysis of exosome protein marker (CD63). CANX is a negative control. (**d-e**) The structure of exosomes were observed using TEM. (**f**) The relationship between miR-133a and FOXL2. (**g**) The expression of miR-133a in endometrial cancer cells. (**h**) The expression of miR-133a in endometrial cancer cell-derived exosomes.

Cancer progression is always related to the microenvironment. Recently, exosomes have been found to modulate cancer cell communication as a novel way and almost all types of cancer cells can release exosomes into their microenvironment^10^. In this study, we hypothesized that endometrial cancer cells could secrete exosomes and pass them on to recipient cells, thereby promoting cancer development. Consequently, we isolated exosomes from the culture supernatants of Ishikawa and HEC-1-A cells (Fig. 8a). The concentration and particle distribution of exosomes were first characterized using NTA analysis, and this revealed average sizes of 127.2 nm and 146.9 nm for Ishikawa and HEC-1-A, respectively (Fig. 8b). Results from Western blotting analysis showed presence of CD63, an exosome marker, in tumor cell-derived exosomes. Conversely, no endoplasmic reticulum protein CANX was detected in the exosomes (Fig. 8c). TEM results showed that the vesicles from endometrial cancer cells were either round or oval, with a complete lipid bilayer membrane structure (Fig. 8d,e). Previous studies have demonstrated presence of miRNAs in exosomes, which are the main source of circulating miRNAs^24^, and play a vital role in cancer development^25^. In this study, we observed that FOXL2 was down-regulated in endometrial cancer tissues and was significantly associated with endometrial cancer. Previous studies have shown that FOXL2 is regulated by miR-133a^26^ (Fig. 8f). Based on this, we detected the expression of miR-133a in all three cell lines. Compared with normal endometrial cancer cells, we found that miR-133a was highly expressed in endometrial cancer cells (Fig. 8g). At the same time, we also detected miR-133a expression in Ishikawa and HEC-1-A-derived exosomes (Fig. 8h). In addition, exosomes derived from endometrial cancer cells could be taken up by normal endometrial cells (Figure S), indicating that endometrial cancer cells release exosomes, which might be then transported to normal endometrial cells.

## Discussion

Endometrial cancer occurs due to abnormal expression of many cancer-related genes^27^, many of which are associated with the susceptibility and development of endometrial cancer^22,23^. Numerous molecular approaches have been performed to identify biomarkers for endometrial cancer^28^. Nonetheless, most of them only focused on a single genetic aspect, limiting the reliability of the biomarkers^29^. In the present study, we used three GEO datasets to perform an integrative analysis with a view of identifying DEGs between normal and cancerous endometrial tissues. The results were validated using the TCGA database. Key biological processes and pathways based on the DEGs between endometrial cancer and normal tissues were also identified. Particularly, we focused on the common DEGs across the three GEO datasets as well as those that were significantly expressed in each dataset. Moreover, we identified exosomes derived from endometrial cancer cells.

Activation of cell proliferation process and p53 signaling pathways have been previously associated with cancers development^30,31^. In this study, GO and KEGG analysis showed that DEGs are involved in progression of endometrial cancer through these biological processes and pathways. A total of 12 DEGs, includingHAND2-AS1, PEG3, OGN, SFRP4, TFAP2A, MMP12, TOP2A, ASPM, CFB, WT1, FOXL2, and EFEMP1, were identified as candidate genes for endometrial cancer. Furthermore, a cross comparison of common DEGs across the three datasets revealed that TOP2A, ASPM, FOXL2 and EFEMP1 were potential biomarkers for distinguishing endometrial cancer, and this was significantly associated with the survival ratios among patients. Further validation in endometrial cancer cells showed that indeed TOP2A, ASPM, FOXL2 and EFEMP1 were significantly regulated. Particularly, TOP2A was significantly up-regulated in endometrial cancer tissues and cells, consistent with Lapinska-Szumczyk *et al*.^32^. These results further indicated that high levels of TOP2A can lead to EC progression. Previous studies have reported that aberrant expression of TOP2A is related to the stage of endometrial cancer and its progression^33^. Functionally, TOP2A encodes a DNA topoisomerase that controls and alters the topologic states of DNA during transcription^34^, and is involved in processes such as chromosome condensation, DNA transcription and replication^35^. High levels of TOP2A have also been detected in other types of cancers, including ovarian cancer^36^ and prostate cancer^37^. Based on these results, therefore, we hypothesized that abnormal expression of TOP2A could be a positive tumor metastasis marker and a poor biomarker for prognosis. In addition, inhibition of TOP2A has been found to mediate a decrease in migration, proliferation or invasion of cells^38^. These results might lead to development of a new treatment strategy for endometrial cancer. Abnormal spindle-like microcephaly-associated (ASPM), which encodes the ASPM protein, plays an essential role in mitotic spindle function during cell replication^39^. ASPM is associated with cell cycle and mediates the tightly coordinated Ubiquitin-Cyclin E-Retinoblastoma-E2F bistable-signaling pathway to control restriction point progression and stem cell maintenance^40^. Previous studies have demonstrated that overexpression of the ASPM gene is associated with progression and poor outcomes in various types of cancer, such as bladder cancer^41^, prostate cancer^42^, and breast cancer^43^. However, none of these studies have elucidated the relationship between ASPM and endometrial cancer. In this study, our results first showed that high levels of ASPM were related to endometrial cancer. When combined with the function of the gene, ASPM is expected to become a new molecule for detection and treatment of this disease although its mechanism in endometrial cancer is yet to be studied.

In addition to the above findings on DEGs, EFEMP1 and FOXL2 were found to be down-regulated in endometrial cancer tissues. The epidermal growth factor-containing fibulin-like extracellular matrix protein 1(EFEMP1), functions as an oncogene or a suppressor, depending on the types of tumor. With previous studies showing that EFEMP1 is a new candidate tumor suppressor gene in endometrial carcinoma^44^. Our findings showed that EFEMP1 was down-regulated in endometrial cancer tissues, as well as endometrial cancer cell lines. Functionally, EFEMP1 inhibits migration of tumor cells by regulating MMP2 and MMP9 via ERK1/2 activity^45,46^. The findings on EFEMP1 in endometrial cancer revealed that the gene could inhibit tumor growth and invasion both *in vitro* and *in vivo* consistent with what has been reported in other types of tumors^44^. A separate studies found that EFEMP1 was elevated in highly invasive ovarian cancer, compared with the low invasive types^47^. Combined, these studies suggest that EFEMP1 plays different roles in the development of different types of carcinoma. FOXL2 on its part encodes a fork head transcription factor. Although many RNA-sequencing studies have been performed to understand the genetic regulation of endometrial cancer, the relationship between FOXL2 and the disease is still lacking^48^. In the current study, analysis on GSE115810 showed that FOXL2 was one of the top10 DEGs found to be down-regulated during the development of endometrial cancer. On the other hand, we observed a significantly low FOXL2 expression in endometrial cancer relative to normal cells. A related study showed that that FOXL2 could suppress cells proliferation and enhance cells apoptosis in cervical squamous cancer, mainly by decreasing Ki67 expression^49^. Various conclusions have been drawn regarding the mechanism of action of FOXL2 in other types of tumors^50^. However, its function remains unclear in endometrial cancer thereby necessitating the need for further clarification using molecular experiments.

A vast array of researches have demonstrated that almost all types of cells can release exosomes that mediate the cell-cell communication^51^, especially tumor cells^52^. Exosomal cargos, particularly those associated with development of cancer, have been considered the best biomarkers for non-invasive diagnostic^53^. Furthermore, studies have revealed that miRNAs are mainly packaged in exosomes. In our study, results from the GO analysis showed that the DEGs were associated with extracellular exosomes. Here, we assumed that the progression of endometrial cancer was mediated by exosomal miRNAs derived from cancer cells. Meanwhile, we observed a down-regulation of FOXL2 in endometrial cancer cells while the expression of miR-133a, which targets FOXL2^54^, was high in the cancerous cells. We found that endometrial cancer cells could secrete exosomes, which contained miR-133a. Exosomal miR-133a may regulate the down-regulation of FOXL2 in endometrial cancer tissues. Furthermore, the progression of endometrial cancer was not only achieved by malignant epithelial cells, but also through involvement of stromal cells. Previous research evidence indicated that extracellular vesicles mediate he communication between tumors and stroma^55^. In the current study, it was found that exosomes derived from endometrial cancer cells could be taken up by stromal cells, indicating that exosomal miRNAs may contribute to the progression of endometrial cancer.

In summary, four core genes (TOP2A, ASPM, EFEMP1 and FOXL2) and several interesting pathways involved in endometrial cancer were identified. Analysis of qRT-PCR assay revealed that expression of mRNA for TOP2A and ASPM are up-regulated in endometrial cancer cells, whereas those of EFEMP1 and FOXL2 are down-regulated. Currently, there are no studies on the role of ASPM and FOXL2 in endometrial cancer. Based on the findings of this study, we propose that multiple molecules can be effectively used to diagnose endometrial cancer. Accordingly, further studies into the functions of these genes can guide development of treatment strategies for EC. In addition, exosomes secreted by endometrial cancer cells, contain miRNAs that regulate FOXL2 and can be transported to normal stromal cells. These results open up new frontiers for the development of approaches for detection and treatment of endometrial cancer. However, further studies are required to elucidate the function and underlying mechanisms of action of these potential biomarkers in the progression of endometrial cancer.

## Materials and Methods

### Study design

The aim of this study was to identify core genes involved in the development of endometrial cancer, using bioinformatics analysis on GEO datasets. A flow diagram of the study design is shown in Fig. 1a. Initially, three GEO datasets (GSE17025, GSE63678 and GSE115810) were reasonably searched in published databases, and then bioinformatics tools used to screen DEGs in the datasets. Analyses on the functions of GO and KEGG pathways were also performed on the DEGs. Next, Genes co-expressed in the three datasets were identified and verified in TCGA database. Survival analyses were performed to further identify core genes related to endometrial cancer. Finally, significant co-expressed genes were verified using endometrial cell lines and miRNAs in exosomes from supernatants of endometrial cancer cell culture evaluated.

### Gene expression omnibus datasets

We retrieved endometrial cancer gene expression array datasets by the GO (https://www.ncbi.nlm.nih. gov/gds) which is a public repository at the National Center of Biotechnology Information for storing high-throughput gene expression datasets^56^. The terms “endometrial cancer” and “Homo sapiens” and “gene expression array” were used to search the potential datasets. Finally, three datasets (GSE17025, GSE63678 and GSE115810) were found suit for our study. Those three datasets contain endometrioid adenocarcinoma tissues and normal endometrial tissues, and the three mRNA chips were sequenced from the same chip company. The detail information of datasets as follows. GSE17025 contained 12 normal endometrial tissues and 79 endometrioid cancer samples^22^. Platform information: GPL570 [HG-U133_Plus_2] Affymetrix Human Genome U133 Plus 2.0 Array. GSE63678 contained 5 normal endometrial tissues and 7 tumor tissues^57^. Platform information: GPL571 [HG-U133A_2] Affymetrix Human Genome U133A 2.0 Array. GSE115810 contained 3 normal endometrial tissues and 24 tumor tissues^58^. Platform information: GPL96 [HG-U133A] Affymetrix Human Genome U133A Array. Those three datasets were used for further analysis.

### Identification of differentially expressed genes

Expression matrixes and platform information were downloaded from GEO database. Then, the datasets were normalized before analysis. R basic (version 3.6.0, https://www.rproject.org/) and Bioconductor packages (available online: http://www.bioconductor.org/) were applied to screen the DEGs^59^. The analysis was performed using limma package at cut off values |log FC | > 1.0 and Adjust P value value <0.05 in these three datasets. Based on the DEGs in each GEO dataset, gene ontology (GO) was used to analyze in terms of biological functions. Kyoto Encyclopedia of Genes and Genomes (KEGG) analysis was employed for pathway analysis. The top 10 analysis results were shown. The GO and KEGG analysis were performed by an online tool DAVID (https://david.ncifcrf.gov/home.jsp)^60^. We used the latest updated data from this site. FDR < 0.05 was considered to indicate a statistically significant difference. VENNY2.1 (https://bioinfogp.cnb.csic.es/tools/venny/index.html) was used to compare the DEGs in three datasets.

### The gene expression in TCGA database and survival analysis

In order to further validate the expression of co-expressed DEGs, the GEPIA (Gene Expression Profiling Interactive Analysis)^61^ database (http://gepia.cancer-pku.cn/) were employed, which based on the TCGA database. Furthermore, we analyzed the correlation between gene expression and survival conditions using TIMER (Tumor IMmune Estimation Resource) (https://cistrome.shinyapps.io/timer/)^62^.

### Cell culture

Ishikawa, HEC-1-A and 4003 cell lines were purchased from cell bank of Chinese Academy of Science (Shanghai, China). Ishikawa and 4003 cells lines were cultured in DMEM (Gibco) culture medium supplemented with 10% FBS. while HEC-1-A cell lines were cultured in McCOY’s 5 A (SIGMA) supplemented with 10% FBS. The cultures were maintained n at 37 °C, under 5% CO2. The two types of endometrial cancer cell lines were derived from adenocarcinoma^63,64^.

### Isolation and identification of exosomes from endometrial cancer cells culture medium

To purify exosomes from tumor cell culture medium, cells were cultured in medium supplemented with 10% exo-free FBS and cell culture supernatants were collected 24–48 h after culture. A standard differential centrifugation protocol was used to isolate exosomes. Briefly, cell cultures were centrifuged at 500 g for 10 min to remove dead cells, followed by a second centrifugation at 2,000 g for 20 min to remove cell debris. Micro and macro vesicles were depleted by centrifugation at 10,000 g for 45 min. Collected supernatants were then centrifuged at 120,000 g for 1.5 h at 4 °C. The pelleted exosomes were resuspended in PBS and washed again, and re-ultracentrifuged at 120,000 g for 1.5 h to collect exosomes.

To study the characteristics of exosomes, transmission electron microscopy was performed. Summarily, exosomes were suspended in PBS and placed onto formvar carbon-coated coper grids at room temperature for 1 min. The exosomes were then stained by 2% uranyl acetate at room temperature for 1 min. and the grids were dried. The stained exosomes were visualized under a TecnaiG2 Spirit120KV transmission electron microscope operating at 120 kV (Thermo FEI).

Particel sizes distribution and concentration of exosomes were examined by Nanoparticle Tracking Analysis (NTA) analysis using a ZetaView PMX 100 (Particle Metrix, Meerbusch, Germany). For each condition analysis, at least three independent experiments were performed.

A western blot analysis was performed to detect exosomal surface protein markers. The exosomal proteins were separated using 10% SDS PAGE, and then transferred onto the polyvinylidene difluoride membranes (Millipore). The membranes were blocked with QuickBlockTM western buffer (Beyotime, China) for 20 min, and incubated with primary antibody(diluted with manufacturer’s instructions) at 4 °C for overnight, followed by a 2 h incubation with HRP-conjugated secondary antibodies at room temperature. The membranes were detected with ECL reagents (Beyotime, China). CD63 was exosome protein marker. CANX, an endoplasmic reticulum protein, was used as the negative control.

### Exosomes tracking analysis

Here, 50 μg of exosomes from supernatants of cultured endometrial cancer cells were incubated for 15 min at room temperature with 20 μM DiI dye containing 5% BSA, added to avert over staining. Exosomes were diluted with PBS and then centrifuged at 120,000 g for 1.5 h at 4 °C. The labeled exosomes were suspended in PBS and collected by ultracentrifugation at 120,000 g for 1.5 h. Next, the labeled exosomes were added to endometrial stroma cell culture medium and incubated for 12 h. The cells were fixed with 4% paraformaldehyde for 20 min, permeabilized with 0.1% Triton X-100 for 15 min, then incubated for 50 min with a FITC-conjugated phalloidin (Solarbio, China) to stain F-actin. DAPI was used to label cell nuclei (Solarbio, China). The respective images were visualized by a confocal microscopy and captured using ZEN lite software (https://www.zeiss.com.cn/microscopy/products/microscope-software/zen.html#inpagetabs-5).

### RNA extraction and qRT-PCR analysis

Total RNA was isolated using the Trizol reagent (TIANGEN, China), then reverse-transcribed to cDNA using the first strand cDNA systhesis kit (TIANGEN, China). To analyze mRNA expression, the SuperReal PreMix Color (SYBR Green) qRT-PCR kit (TIANGEN, China) was used, with GAPDH as an internal amplification control. We isolated cellular and exsomal miRNAs using miRNeasy Mini Kit (QIAGEN, Germany), and performed cDNA synthesis using miRcute Plus miRNA First-Strand cDNA Kit (TIANGEN, China). miRNA expression was examined using miRcute Plus miRNA qPCR Kit (SYBR Green) (TIANGEN, China). To detect cellular miRNAs, U6 was used as a standard control. To compare the expression of miRNAs between cell and exosomes, equivalent synthetic external control cel-39–3p was added to the system during the reverse transcription step. Primer sequences used during PCR analysis are outlined in Table S1.

### Statistical analyses

Data were presented as mean ± standard errors of the means. A student’s t-test was used to reveal any significant differences between two groups, while One-way analysis of variance (ANOVA) was performed to compare multiple (>2) means. Statistical analyses were performed using SPSS 22.0 software. P-values ≤ 0.05 were considered to be significant. All experiments were performed using at least 3 independent replicates.

## Data availibility

The data that support the findings of this study are available in GEO at https://www.ncbi.nlm.nih.gov/geo/. These data were derived from the following resources available in the public domain: GSE17025, GSE63678 and GSE115810. The authors declare that there are no conflicts of interests. The corresponding author is responsible for submitting a competing interest’s statement on behalf of all authors of the paper.

## Supplementary information

Supplementary Information.
