# Supplementary material for: Identification of core genes in the progression of endometrial cancer and cancer cell-derived exosomes by an integrative analysis
Source: Sci Rep. 2020 Jun 17;10:9862. doi: 10.1038/s41598-020-66872-3 (PMC7299953; doi:10.1038/s41598-020-66872-3)
Supplement: Supplementary file 1 — Supplementary Information. [file 41598_2020_66872_MOESM1_ESM.docx]

**Identification of core genes in the progression of endometrial cancer and cancer cell-derived exosomes by an integrative analysis**

Shuang Shi^1+^, Qiang Tan^1+*^, Fuqiang Feng^2^, Heping Huang^1^, Jingjie Liang^1^, Dingren Cao^1^, Zhengguang Wang^1*^

1 College of Animal Sciences, Zhejiang University, Hangzhou, Zhejiang, P. R. China

2 Agricultural Economic Service Center of Wuzhen Town, Tongxiang, Zhejiang, P. R. China

^*^Corresponding. wzhguang68@zju.edu.cn and tq19952010@126.com

^+^these authors contributed equally to this work

| **Symbol** | **Sequences** |
| --- | --- |
| TOP2A | F: ACCATTGCAGCCTGTAAATGA  R: GGGCGGAGCAAAATATGTTCC |
| ASPM | F: TGCAGTGGGTGAACATGAAAA  R: CGAAGAGGGTGTTACCTCGTTT |
| FOXL2 | F: GGTCGCACAGTCAAGGAGC  R: CGCGATGATGTACTGGTAGATG |
| EFEMP1 | F: CAAAGTGAACACAACGTGTGC  R: TGCTCCCCTCGCTTCTGAT |
| GAPDH | F: ACAACTTTGGTATCGTGGAAGG  R: GCCATCACGCCACAGTTTC |
| MiR-133a | TTTGGTCCCCTTCAACCAGCTG |
| cel-39-3p | TCACCGGGTGTAAATCAGCTTG |
| U6 | AACGAGAAGCGAACCAAAAAAA |

Table S1. The information of primers used in thus study.


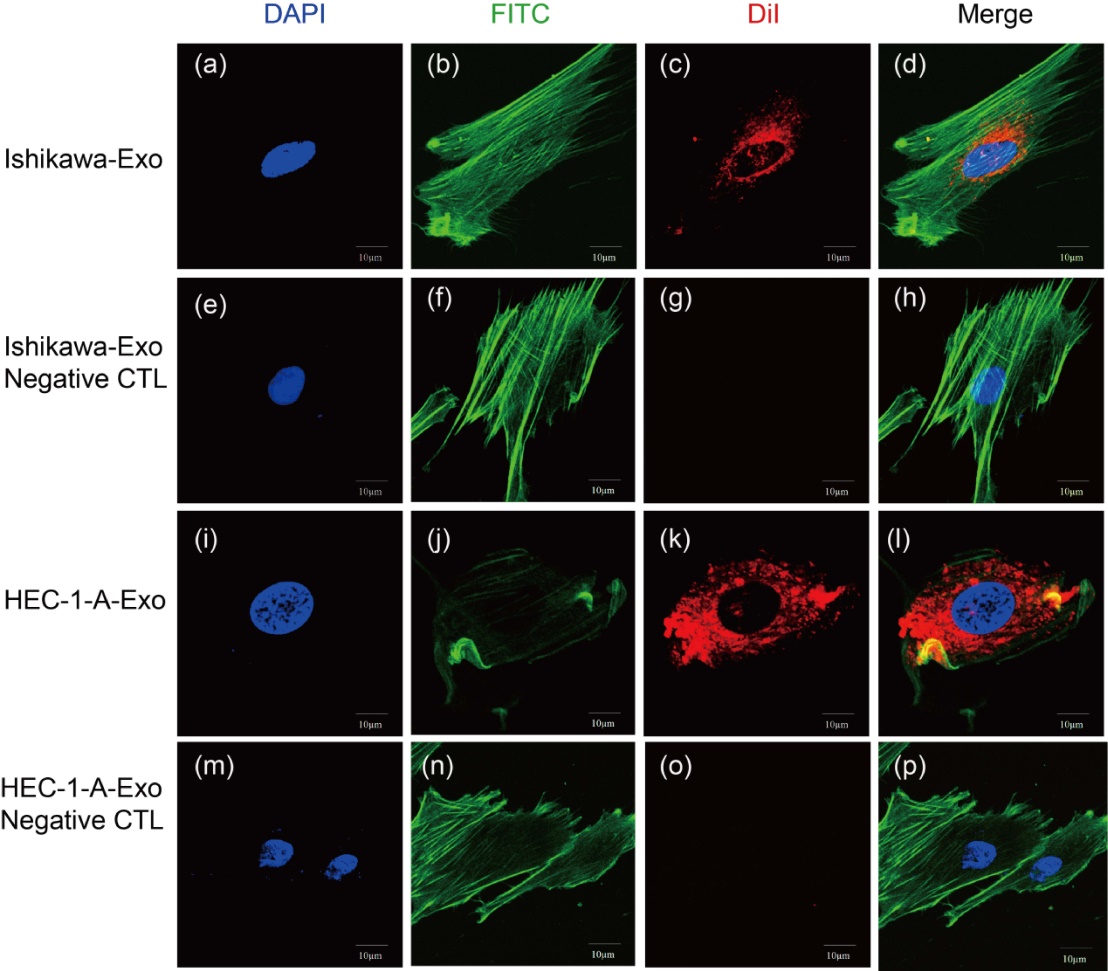


**Figure S**. In vitro model of endometrial cancer cells-stromal cells communication. Exosomes derived from endometrial cancer cells (a-d, Ishikawa. i-l, HEC-1-A) were internalized by normal endometrial cell. e-h and m-p as negative control. Nuclei stained by DAPI in blue, exosomes stained by DiI in red and actin stained by FITC-conjugated phalloidin in green. Bar=10μm.

Supplementary Figure S1 continued (Figure 8c)


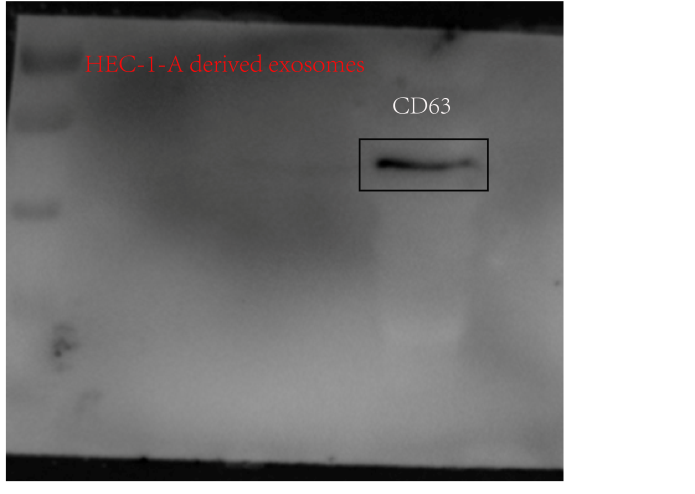


36KD


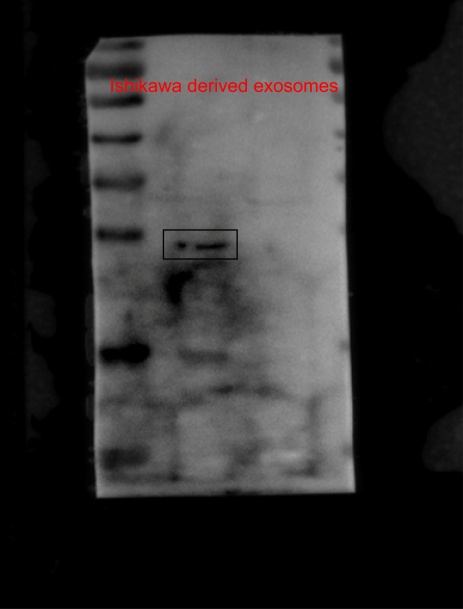


36KD


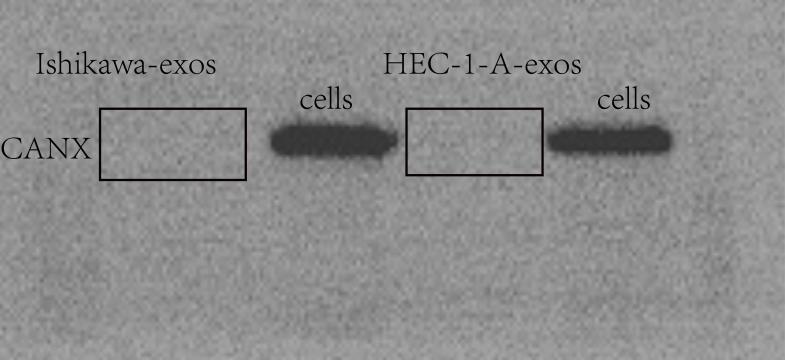


90KD

Figure S1. Full, uncropped images of all immunoblots shown in the main figures,

as well as supplementary figures. Molecular weight markers are indicated on the right of all blots.
